# Mechanical Properties and Material Characteristics of 3D-Printed Titanium Capsules for Cancer Drug Delivery Applications

**DOI:** 10.3390/ma18132969

**Published:** 2025-06-23

**Authors:** Katarzyna Kazimierska-Drobny, Grzegorz Szala, Janusz Musiał, Marek Macko, Tomasz Karasiewicz, Jakub Lewandowski

**Affiliations:** 1 Faculty of Mechatronics, Kazimierz Wielki University, Mikolaj Kopernik 1 Street, 85-074 Bydgoszcz, Poland; gszala@ukw.edu.pl (G.S.); janusz.musial@ukw.edu.pl (J.M.); mackomar@ukw.edu.pl (M.M.); jlewy@ukw.edu.pl (J.L.); 2 Faculty of Material Engineering, Kazimierz Wielki University, Mikolaj Kopernik 1 Street, 85-074 Bydgoszcz, Poland; tomakara@ukw.edu.pl

**Keywords:** 3D-printed titanium capsules, Ti-6Al-4V, DMLS, biomedical implants

## Abstract

The aim of the study was to assess the mechanical and material properties of porous titanium capsules, produced by 3D printing via the DMLS (Direct Metal Laser Sintering) technique based on their potential application as carriers for anticancer drugs. The study used capsules made from the Ti-6Al-4V alloy, and analyzes the impact of geometric parameters, structural features, and printing angles (0°, 45°, and 90°) on their compressive strength. A total of 36 capsules were tested, 18 of type KTD and 18 of type KTM, each in two loading directions. The surface roughness and damage characteristics resulting from mechanical loading have also been evaluated. Statistical analysis of the results was performed using Student’s *t*-test. The results show that the capsules printed at an angle of 45° are characterized by the highest compressive strength, while their resistance significantly exceeds the values typical of human bone tissue. Additionally, the observed damage does not lead to the formation of sharp edges or loose fragments, which confirms the safety of their use in the body. The high surface roughness promotes tissue integration and limits capsule migration after implantation. The analyses confirm the potential of 3D-printed titanium capsules as effective and safe drug carriers in personalized anticancer therapy.

## 1. Introduction

The design and fabrication of porous titanium capsules is gaining increasing interest in biomedical applications, especially as carriers of anticancer drugs [1,2,3,4,5,6,7]. Porous titanium capsules are particularly effective in drug delivery as their structure enables the controlled release of the active substances, which increases the efficacy of the therapy and minimizes any side effects. Particular attention is also given to the application of porous titanium scaffolds in bone tissue engineering [8,9,10,11,12], where porous they support the bone tissue regeneration by facilitating the ingrowth of bone cells and the formation of blood vessels. Other titanium implants, such as dental and orthopedic implants, also exploit the unique properties of this metal [9,13,14,15,16,17]. Titanium-based implants are widely used due to their capacity for osseointegration, which translates into high durability and long-term stability. In orthopedics, titanium is utilized in endoprosthetic surgery and the reconstruction of bone defects, contributing to reduced patient recovery time.

Additive manufacturing techniques, such as 3D printing, represent a key technology in the fabrication of porous titanium structures [8,9,10,15,18]. The application of these technologies enables precise control over both the mechanical and structural properties of the produced capsules [14,18,19,20]. In the case of 3D-printed titanium capsules, this enhances the functionality of the drug delivery systems, allowing them to be tailored to specific requirements regarding active substance release profiles [21]. The control of such parameters as pore size and distribution is critical to their functional properties, influencing both the drug loading capacity and release kinetics [21].

Moreover, the 3D printing method enables a layer-by-layer construction of complex geometries that are difficult to achieve using conventional manufacturing methods [10,13,14], primarily due to the characteristics of printed titanium. Studies on the influence of print orientation on the mechanical strength of materials, particularly titanium alloys, have demonstrated significant differences in mechanical properties, depending on the angle at which the additive manufacturing process is conducted. Research on the Ti-6Al-4V alloy has shown that the print orientation (vertical vs. horizontal) significantly affects both microstructure and tensile strength, with vertically printed specimens exhibiting superior mechanical performance [22]. Another study demonstrated that varying orientation angles (0°, 30°, 60°, 90°) result in anisotropic material behavior, which is a critical consideration in the context of 3D printing technology [23]. From a design and engineering application perspective, optimizing the printing angle is crucial for obtaining components with high and stable mechanical strength [24]. Studies on titanium have shown that appropriate print orientation can enhance not only its strength but also the material’s wear resistance [25]. Some studies indicate that heat treatment and powder quality may have an impact on mechanical properties comparable to that of print orientation [26]. The porosity distribution also varies depending on the build orientation, with horizontally printed samples often exhibiting higher porosity levels, especially in 45° and 90° orientations, due to incomplete melting of the powder particles [26,27].

High strength combined with low weight, corrosion resistance, and biocompatibility make titanium safe for the human body and do not cause immunological reactions. A key advantage is the ability to fabricate precisely controlled porous structures using 3D printing, which enables increased surface area, the adjusting mechanical properties and drug release profiles [1,2]. Porosity facilitates efficient storage and controlled release of the active substances, making titanium-based carriers an ideal solution for anticancer therapies and personalized drug delivery systems [5]. Additionally, 3D printing technology enables rapid prototyping and personalization, allowing drug carriers to be tailored to individual patient needs and facilitating the fabrication of complex geometries that are difficult to achieve with conventional methods. The use of advanced manufacturing techniques for the production of porous titanium capsules not only enhances the efficiency of drug delivery systems, but also supports the development of personalized medical therapies, enabling the adaptation of the treatment to suit individual patient needs [10,14,17,21,28,29,30]. The mechanical properties of porous titanium capsules are closely dependent on their porosity and structural design [17]. Studies have shown that the Young’s modulus and flexural strength of porous titanium can be adjusted to levels comparable to those of human cortical bone, provided the material is fabricated with appropriately selected porosity [14,18]. The possibility of precise control of porosity within the 5.0% to 37.1% range by volume facilitates the adjustment of the mechanical properties to suit the biological requirements, which promotes osseointegration and ensures mechanical stability, especially in implant applications [8,16,17,18].

The aim of this study is to determine the suitability of porous titanium capsules, manufactured using 3D printing, as carriers for anticancer drugs. The research includes an analysis of the structural features of the capsules, such as geometric parameters, material properties, and mechanical response under static loading. The study determines the strength characteristics of capsules made from Ti-6Al-4V titanium alloy, manufactured using the Direct Metal Laser Sintering (DMLS) technique. The influence of porosity and internal structure on the mechanical properties of the capsules was determined, and a surface roughness analysis were also performed. Additionally, an assessment was made of the damage mechanisms resulting from compressive forces. With its focus on the strength, geometrical, and structural evaluations, the research aims to assess the design characteristics of titanium capsules to confirm their suitability as safe and effective carriers for anticancer drugs. The objective is to demonstrate that the capsules are sufficiently strong, biocompatible, and have an appropriate structure for controlled drug release within the patient’s body.

This study focuses on the mechanical and morphological characterization of porous titanium capsules intended for use in personalized drug delivery. Unlike many existing works that focus on drug release modeling or in vitro testing, our approach emphasizes the optimization of 3D printing parameters—such as build orientation and geometry—and their impact on structural integrity. The novelty lies in experimentally linking print angle, capsule design, and compressive failure behavior, which are critical factors in ensuring safe and reliable performance of implantable drug carriers.

## 2. Materials and Methods

**Figure 1:**
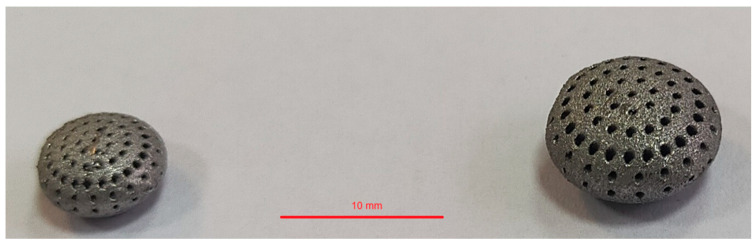
View of 3D-printed Ti-6Al-4V titanium alloy capsules for anticancer drug delivery.

The subject of the study was capsules made of Ti-6Al-4V titanium alloy, fabricated using the DMLS method on an Orlas Creator 3D printer (O.R. Lasertechnologie GmbH, Dieburg, Germany) with the following process parameters: layer thickness of 25 µm, laser power of 82 W, and scanning speed of 500 mm/s (Figure 1). The particle size of the titanium powder used in the DMLS process ranged from 30 to 63 µm. After printing, the capsules were cleaned of residual powder and subjected to sandblasting to remove surface imperfections and standardize surface roughness.

**Figure 2:**
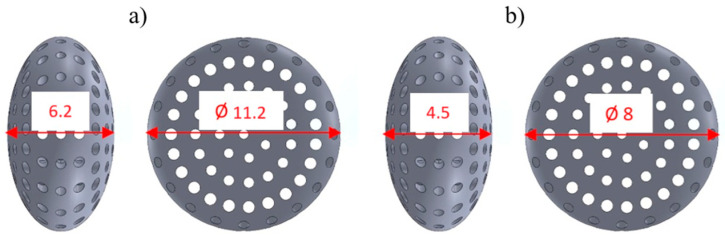
External dimensions of titanium capsules; (**a**) KTD capsule, (**b**) KTM capsule.

Two capsule sizes were examined: the KTD capsule (refers to the larger variant) with the main dimensions of 6.2 mm and 11.2 mm (Figure 2a), and the KTM (refers to the smaller variant) capsule with the dimensions of 4.5 mm and 8 mm (Figure 2b).

The remaining geometrical features of the capsules were as follows: the wall thickness was 1.23 mm for the KTD capsule and 0.85 mm for the KTM capsule. In terms of drug-release hole design, the KTM capsule featured a spacing of 1.0 mm between the struts (holes) with a hole diameter of 0.40 mm, while the KTD capsule had a spacing of 1.3 mm and a hole diameter of 0.50 mm. The measured surface roughness (Rz) of the capsules was 118.9 µm. Surface roughness was measured using a contact profilometer (MC-RT 810) (MC Instrument, Raciborz, Poland) in accordance with ISO 25178-601:2025 [31]. A scan length of 4.8 mm was used. Due to the curvature of the capsule surface, the measurement was conducted on a flat reference specimen printed using the same DMLS process and material as the capsules. The measurement direction was aligned with the build orientation.

The strength tests were performed using an Instron 3367 two-column testing machine (Instron, Norwood, MA, USA) with a mechanical drive, controlled by a computer equipped with Bluehill 2 software. This facilitates the application of tensile or compressive forces up to a maximum of 30 kN, with a continuously adjustable loading rate ranging from 0 to 500 mm/min.

The compressive tests were carried out at a constant loading rate of 1 mm/min. All measurements were performed under standard laboratory conditions (approximately 20 °C, 50% relative humidity). Preloading was not applied. Given the experimental design and non-standard capsule geometry, the procedure did not follow ISO or ASTM testing protocols.

The structural examination of the external surfaces of KTD and KTM capsules was carried out using a Mitutoyo QV Apex coordinate measuring machine (Mitutoyo, Tokyo, Japan), which was used to capture detailed images of the capsule surface layers. Additionally, images of damage sites after compression testing were taken to assess the size, location, and type of failure. Micro-CT imaging was performed using a SkyScan 1270 system (Bruker, Billerica, MA, USA). Image reconstruction and analysis were carried out using NRecon–version 1.7.4.2, CTan–version 1.18.8.0+, and CTvox software–version 3.3.0r1403 (provided by the manufacturer). Scanning was conducted at 80 kV source voltage and 50 µA current. Depending on the sample, voxel size was either 7.50 µm or 8.00 µm.

## 3. Results

### 3.1. Results of Mechanical Testing

**Figure 3:**
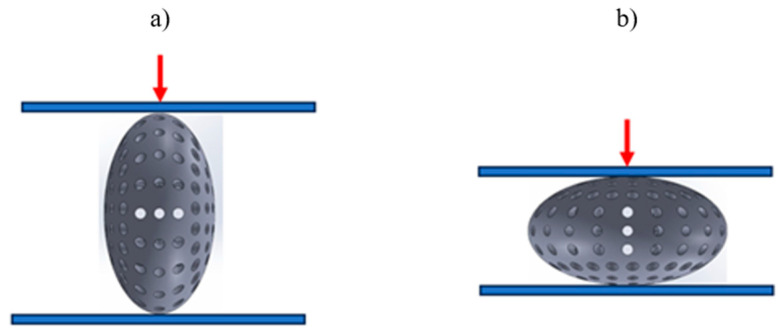
Direction of compressive force application: (**a**) parallel to the longitudinal axis, (**b**) parallel to the short axis of the capsule.

Compression tests were conducted on both KTD and KTM capsules under two loading directions: parallel to the capsule’s longitudinal axis (Figure 3a) and parallel to the short axis (Figure 3b).

**Figure 4:**
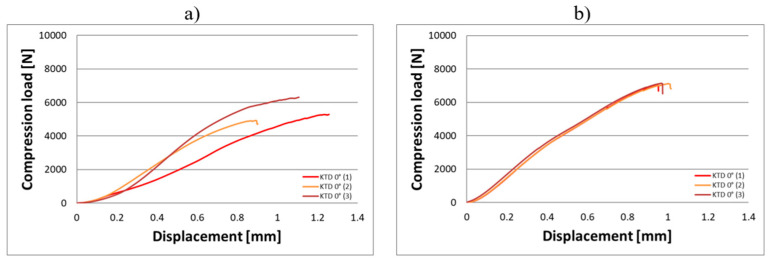
Load–displacement graph from compression testing of the KTD capsule printed at 0°, oriented for the compressive force applied, (**a**) along the longitudinal axis, (**b**) along the short axis.

**Figure 5:**
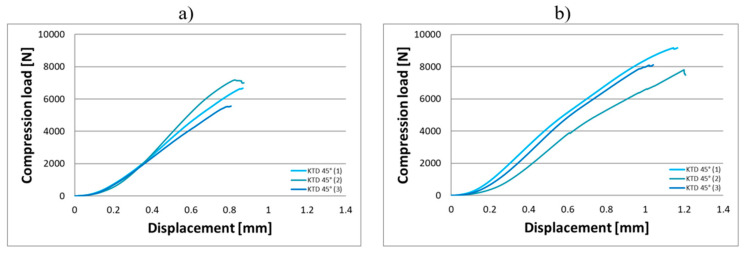
Load–displacement graph from compression testing of the KTD capsule printed at 45°, oriented for the compressive force applied; (**a**) along the longitudinal axis, (**b**) along the short axis.

**Figure 6:**
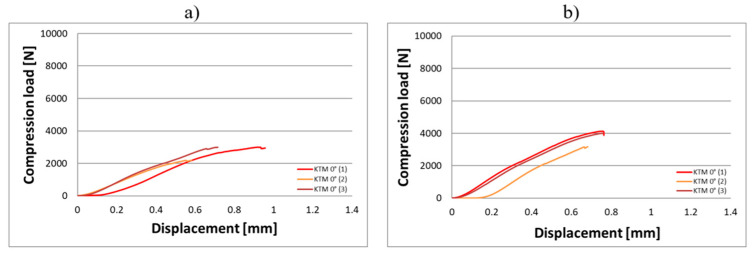
Load–displacement graph from compression testing of the KTM capsule printed at 0°, oriented for the compressive force applied: (**a**) along the longitudinal axis, (**b**) along the short axis.

**Figure 7:**
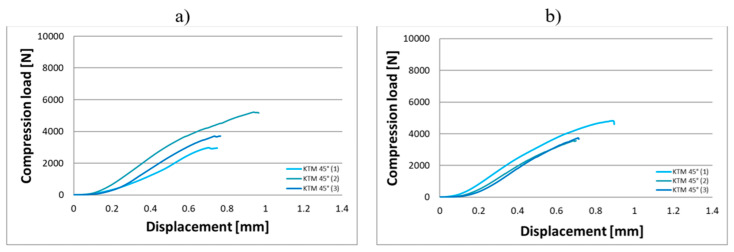
Load–displacement graph from compression testing of the KTM capsule printed at 45°, oriented for the compressive force applied: (**a**) along the longitudinal axis, (**b**) along the short axis.

Compression tests were conducted on eighteen KTD and eighteen KTM capsules, each tested in two orientations relative to the direction of the applied compressive force, in the horizontal and vertical positions (Figure 3). The capsule print orientation was also considered during fabrication, being printed at angles of 0° (horizontal printing), 45°, and 90°. Based on the results obtained from the strength tests, force–displacement curves were developed for the larger KTD capsules (Figure 4 and Figure 5) and the smaller KTM capsules (Figure 6 and Figure 7). Additionally, mechanical parameters were calculated, including the average force at permanent capsule failure, the average displacement at maximum compressive load, and the standard deviation.

The test results for the KTD capsules are given in Table 1 and for the KTM capsules in Table 2. Capsules printed at 90° were excluded from the results, as their strength was found to be comparable to those printed at 0°.

Statistically significant differences for the parameter Maximum Compressive Load (N) were observed only in the comparison between 0° and 45° print orientations for the short axis, with a *p*-value of 0.030 using Student’s *t*-test (test power: 70%).

A significant difference was also found for Displacement at Maximum Compressive Load (mm) in the case of the short axis, where the *p*-value was 0.021 (test power: 79%).

In all other cases, no statistically significant differences were observed.

In the comparisons performed using Student’s *t*-test, no statistically significant differences were found between the 0-degree and 45-degree print orientations for either the short or long axis, with respect to the parameters Maximum Compressive Load (N) and Displacement at Maximum Compressive Load (mm).

**Figure 8:**
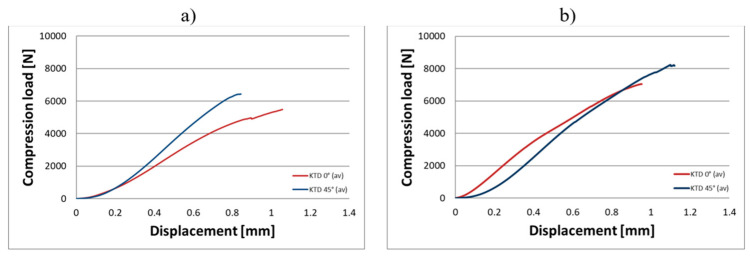
Comparison of average compressive loads for KTD capsules, depending on printing angle; 0° (brown) and 45° (blue): (**a**) KTD—load applied along the longitudinal axis; (**b**) KTD—load applied along the short axis.

**Figure 9:**
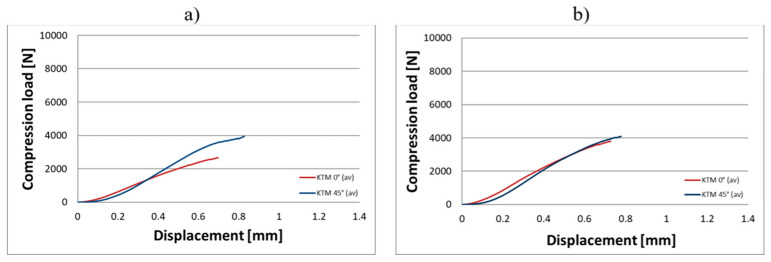
Comparison of average compressive loads for KTM capsules depending on printing angle; 0° (brown) and 45° (blue): (**a**) KTM—load applied along the longitudinal axis; (**b**) KTM—load applied along the short axis.

The analysis of the compression test results indicates that the proposed titanium capsules exhibit sufficient maximum compressive force for use as implantable containers for controlled drug release in human tissue. The average compressive strength for KTD capsules printed at 0° was 5460.15 N (load applied along the longitudinal axis) and 7103.01 N (load applied along the short axis), while for KTM capsules printed at the same angle, it was 2674.81 N (longitudinal axis) and 3757.31 N (short axis). These values demonstrate that the selected material Ti-6Al-4V titanium alloy possesses significantly higher strength than that of the human skeletal, muscular, and soft tissue systems, and is capable of withstanding loads resulting from various types of accidents, such as impact, or crushing. Higher compressive strength was observed for capsules printed at a 45° angle: 6433.91 N (load applied along the longitudinal axis) and 8416.92 N (short axis) for the KTD capsules, and 3967.43 N (longitudinal axis) and 4047.99 N (short axis) for the KTM capsules. The differences between capsules printed at 0° and 45° are illustrated in the graphs for average compressive loads concerning KTD (Figure 8) and KTM (Figure 9).

### 3.2. Characterization of Titanium Capsule

**Figure 10:**
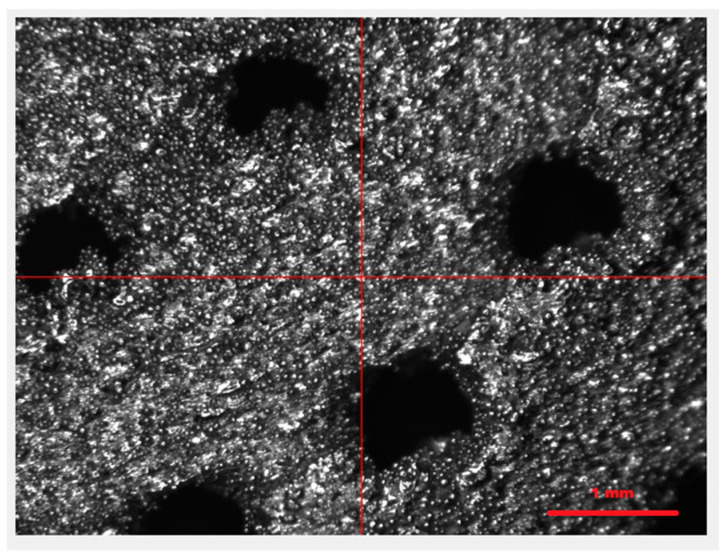
External structure of a 3D-printed capsule made of Ti-6Al-4V titanium alloy.

**Figure 11:**
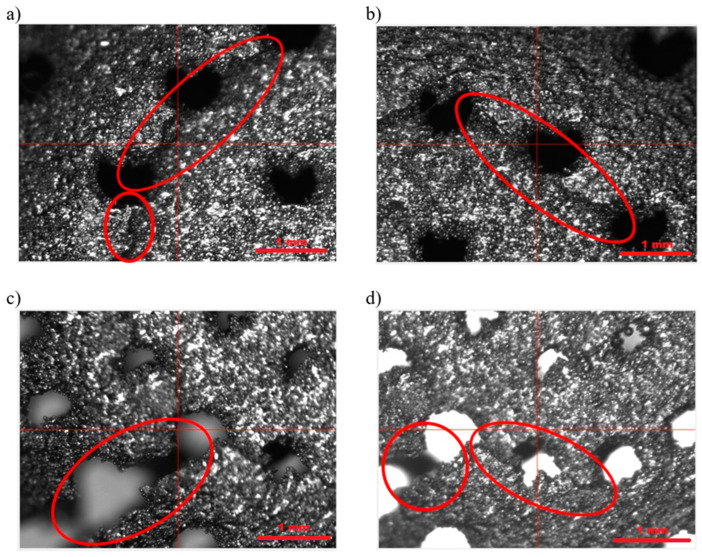
Structure of titanium capsule walls after compression testing, showing visible mechanical damage. Images (**a**–**d**) correspond to different views of the measurement sites.

**Figure 12:**
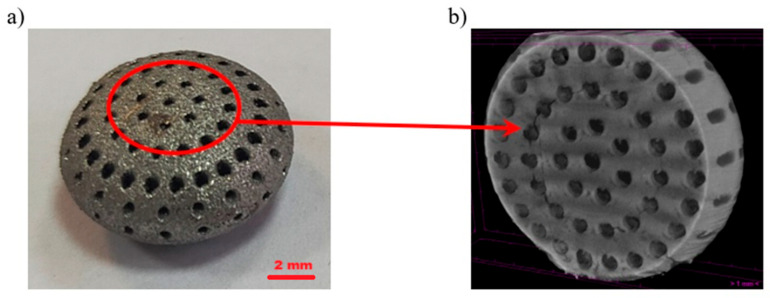
Capsule damage line under compression: (**a**) visible fracture line, (**b**) CT scan image indicating the damage line.

Based on the conducted external and internal structural analyses, a total of 20 photographs were obtained for these purposes. Representative images of the external structure and damage resulting from compression are presented in Figure 10, Figure 11 and Figure 12.

Figure 10 shows the structure of the capsule. The clearly visible rough surface (with a measured surface roughness Rz of 118.9 µm) is the result of the manufacturing process. The capsules were fabricated using the DMLS method, i.e., an additive technique, in which titanium powder is melted layer by layer using a laser. As seen in the images (Figure 10 and Figure 11), the titanium powder was melted incompletely. Unmelted powder grains are clearly visible on the surface. These residual particles contribute to the high surface roughness of the final product, giving the capsule a porous-like appearance. This geometric surface feature is advantageous for capsules implanted into the human body, as it significantly reduces uncontrolled migration of the implant and promotes better assimilation with human tissue compared to smooth-surfaced materials. Figure 11 presents the damage resulting from the compression strength test. The damage occurred in the areas indicated in Figure 12, where the compressive force was applied along the short axis of the capsule. The nature and location of the damage at the macro scale (Figure 12) indicate cracking of the structure between the drug-release openings, which is to be expected. At the micro-scale (Figure 11), it can be observed that the cracks propagate between unmelted titanium powder grains, without the presence of loose material fragments—an advantageous outcome, as it eliminates the risk of debris that could pose a threat to the body. Additionally, the intergranular cracks do not result in the formation of sharp edges that could damage surrounding tissue, which further supports the safety of the capsule design for biomedical applications.

## 4. Summary and Conclusions

The proposed Ti-6Al-4V titanium alloy is a biomaterial suitable for intra-tissue applications. The use of DMLS 3D printing for capsule fabrication offers several advantages:

- It is a widely adopted method for producing components from titanium powder;
- The resulting surface (high surface roughness, Rz = 118.9 µm) significantly limits uncontrolled migration of the implanted capsules (as no experimental data on capsule migration are available, this remains a preliminary hypothesis pending future validation);
- It provides sufficient compressive force, ranging for KTD capsules from 5460.15 N (capsules printed at 0° and loaded along the longitudinal axis) to 8416.92 N (capsules printed at 45° and loaded along the short axis), and for KTM capsules from 2674.81 N (printed at 0° and loaded along the longitudinal axis) to 4047.99 N (printed at 45° and loaded along the short axis).

The load values presented in the results of this study demonstrate that the compressive force of the capsules significantly exceeds the strength of the human skeletal, muscular, and soft tissue systems.

The damage caused by crushing the titanium capsule proposed in the publication limits the complications caused by the above event. They do not cause capsule fragmentation (do not generate loose elements) and the resulting edges do not cause tissue damage (most of them have rounded edges—submerged grains of titanium powder in a spherical shape). Cracks occur between grains of titanium powder, which minimizes the risk of sharp edges that can damage the tissue. The nature of the damage indicates cracking of the structure between the holes intended for drug application, which is in line with the expectations.

Roughness at the level of Rz = 118.9 μm is beneficial because it limits the movement of implanted capsules and improves their assimilation with the tissue.

The DMLS printing method facilitates the precise control of the mechanical and structural properties of the capsules. The capsules can be tailored to the individual needs of patients, which is crucial in personalized therapy. Three-dimensional printing technology facilitates the rapid prototyping and personalization of drug carriers.

The results of the studies presented in the publication and their analysis indicate that 3D-printed porous titanium capsules (Ti4Al6V) form a promising solution in anticancer therapy and can be used as effective and safe drug carriers, with the possibility of adapting them to the individual needs of the patients.

## Figures and Tables

**Table 1:** Strength test results for KTD capsules printed at 0° and 45°.

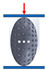	Maximum Compressive Load (N)		Displacement at Maximum Compressive Load (mm)	
	0°	45°	0°	45°
1	5255.98	6571.38	1.22	0.84
2	4881.01	7134.22	0.88	0.85
3	6243.46	5596.12	1.08	0.83
Mean	5460.15	6433.91	1.06	0.84
Standard Deviation	703.798	778.211	0.171	0.010
*p* value	0.183		0.090	
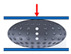				
1	7044	9166.2	0.95	1.14
2	7123.44	7812.83	1	1.2
3	7141.58	8271.72	0.97	1.06
Mean	7103.01	8416.92	0.97	1.13
Standard Deviation	51.900	688.269	0.025	0.070
*p* value	0.030		0.021	

**Table 2:** Strength test results for KTM capsules printed at 0° and 45°.

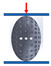	Maximum Compressive Load (N)		Displacement at Maximum Compressive Load (mm)	
	0°	45°	0°	45°
1	2997.61	2974.29	0.92	0.71
2	2120.72	5219.36	0.53	0.94
3	2906.11	3708.63	0.66	0.74
Mean	2674.81	3967.43	0.70	0.80
Standard Deviation	482.035	1144.691	0.199	0.125
*p* value	0.146		0.529	
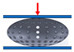				
1	4124.84	4819.17	0.75	0.89
2	3155.02	3581.42	0.66	0.69
3	3992.08	3743.38	0.76	0.72
Mean	3757.31	4047.99	0.72	0.77
Standard Deviation	525.808	672.753	0.055	0.108
*p* value	0.587		0.569	

## Data Availability

The original contributions presented in this study are included in the article. Further inquiries can be directed to the corresponding author.
